# Nimotuzumab combined with radiotherapy for the treatment of hypopharyngeal cancer - A case report from a tertiary cancer center

**Published:** 2021-03-16

**Authors:** Aswin Nagarajan, Arun Sakthivelu, Narendran Santhanaraman, Ramya Ravichandar

**Affiliations:** ^1^Department of Radiation Oncology, Cancer institute, Adyar, Chennai, Tamil Nadu, India; ^2^Assistant Professor, Department of Pharmacology, Sree Balaji Medical College and Hospital, Chennai, India

**Keywords:** Nimotuzumab, carcinoma hypopharynx, complete response, disease-free interval

## Abstract

**Background:**

Nimotuzumab is a monoclonal antibody against epidermal growth factor receptor which can be combined with chemotherapy and radiotherapy for the treatment of locally advanced unresectable squamous cell carcinoma of head-and-neck region and its role has already been established in India.

**Aim:**

The aim of this case report is to show the role of nimotuzumab in carcinoma hypopharynx.

**Methods:**

We report a patient with Stage III carcinoma of hypopharynx, who received radiotherapy along with weekly nimotuzumab due to his comorbidities. The patient had tolerated the treatment very well without any major side effects.

**Results:**

The patient is on regular follow-up with a complete response (CR) of the disease and with the disease-free interval (DFI) of 7 months.

**Conclusions:**

Nimotuzumab along with radiotherapy can be safely given in the patients with carcinoma hypopharynx, who are ineligible for chemotherapy.

**Relevance for patients:**

Nimotuzumab can be added with radiotherapy to the patients with head-and-neck malignancies who are ineligible for chemotherapy to improve the clinical outcome with minimal toxicity.

## 1. Introduction

Squamous cell carcinoma of the head-and-neck region is one of the most common malignancies worldwide accounting for more than 90% of all head-and-neck cancers [1]. Majority of the patients present to the clinician in a locally advanced stage in India and hence the outcomes are poor [2]. The major causative factors are smokeless tobacco and betel nut in Asian population whereas cigarette smoking and alcohol in Western population [3,4]. Surgery is the mainstay of treatment for early stage cancers [5].

For locally advanced unresectable head-and-neck cancers, concurrent chemoradiation, especially with cisplatin, is the preferred treatment [6]. Although it had superior outcomes therapeutically, the survival benefit was low with added toxicities [7]. Hence, additional strategies are needed for improving the outcome of head-and-neck cancers.

More than 80% of the squamous cell cancers of the head and neck have over expression of the epidermal growth factor receptor (EGFR) which correlates with locoregional failure, distant metastases, and poor prognosis [8]. Hence, therapies targeting EGFR have become more popular for the treatment of head-and-neck cancers.

EGFR-targeted therapies such as cetuximab and nimotuzumab have shown improvements in progression-free survival (PFS) and overall survival (OS) in patients with head-and-neck cancers [9]. Cetuximab is a chimeric human/murine anti-EGFR monoclonal antibody (MAb). The EXTREME trial showed that the addition of cetuximab- to platinum-based chemotherapy leads to a prolonged median survival from 7.4 to 10.1 months when compared to chemotherapy alone in recurrent and/or metastatic head-and-neck squamous cell cancers [10]. However, the use of cetuximab is associated with hypersensitivity, severe skin toxicity, gastrointestinal adverse effects, and electrolyte imbalances [11].

Nimotuzumab is a new humanized anti-EGFR MAb that binds to the extracellular domain of the EGFR with intermediate affinity and high specificity which results in the blockade of receptor-dependent signal transduction pathways and provides anti-tumor effects [12]. The advantage of nimotuzumab over other anti-EGFR Mab is its benign adverse effect profile as it requires bivalent binding for stable attachment, leading to selective binding to tumor cells that overexpress the EGFR level. When EGFR expression is low, which is seen in normal tissues, monovalent interaction of nimotuzumab is transient, thus sparing normal healthy tissues and avoiding severe toxicities [13]. This probably explains the lesser toxicities associated with nimotuzumab therapy.

We report a patient with carcinoma of hypopharynx who received radiotherapy along with weekly nimotuzumab due to his comorbidities. The patient had tolerated the treatment very well without any major side effects. He is on regular follow-up with a complete response (CR) of the disease and with the disease-free interval (DFI) of 7 months.

## 2. Case Report

A 57-year-old male smoker and alcoholic with comorbidities of diabetes mellitus, systemic hypertension, and chronic kidney disease presented to our hospital with the complaints of dysphagia and pain on the left side of the neck radiating to the ear for the past 3 months. He was hemodynamically stable. Oral cavity examination was unremarkable. Indirect laryngoscopy revealed a growth in the left lateral pharyngeal wall extending to the left tonsil superiorly and to the left pyriform fossa inferiorly. Neck examination revealed no significant lymphadenopathy.

Blood investigations revealed raised renal function tests and low creatinine clearance. Upper gastrointestinal endoscopy revealed a large ulceroproliferative growth in the left lateral pharyngeal wall extending proximally to the left tonsillar fossa and distally up to the upper one-third of lateral wall of the left pyriform fossa. The esophagus showed a small area of unhealthy mucosa at 31 cm. Narrow band imaging showed the unhealthy area as an erosion due to reflux disease. Biopsy from the hypopharyngeal lesion was suggestive of squamous cell carcinoma Grade II–III. Computerized tomography (CT) revealed a 4.5 × 4 × 2 cm lesion in the left pyriform fossa and left lateral pharyngeal wall. Hence, the patient was diagnosed with carcinoma of hypopharynx T3N0M0 Stage III.

The surgical oncologist opined to proceed with organ preservation approach. The medical oncologist opined to get the nephrologist’s opinion for proceeding with chemotherapy. The nephrologist advised to avoid platinum-based drugs in view of chronic kidney disease. After discussion in the multidisciplinary board, the patient was planned for radiotherapy with concurrent nimotuzumab.

**Figure 1:**
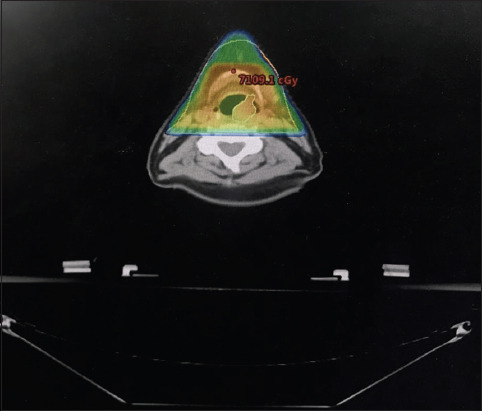
The radiotherapy dose distribution of the patient.

The patient received radiotherapy of a total dose of 66 Gy (200 cGy/fraction up to 33 fractions) using conformal technique (Varian Linear Accelerator) along with 5 cycles of weekly nimotuzumab (200 mg) (Figure 1). During radiotherapy, the patient developed Grade II mucositis, Grade II pharyngitis, and Grade I neutropenia. The patient completed the treatment without much treatment related side effects. Follow-up upper gastrointestinal endoscopy after 6 weeks revealed post-radiotherapy changes with no evidence of disease. The patient is on regular follow-up with a disease-free interval (DFI) of 7 months.

## 3. Discussion

This case report shows the efficacy of concurrent targeted therapy with radiotherapy, for patients ineligible for chemotherapy. This has the advantage of minimal adverse effects in locally advanced, unresectable squamous cell carcinoma of the head and neck. Nimotuzumab has the advantage of not having side effects of other anti-EGFR monoclonal antibodies like cetuximab which can be attributed to its mechanism of action [14].

The efficacy and safety of nimotuzumab in locally advanced unresectable, recurrent and metastatic squamous cell carcinoma of head and neck have been well-established in the literature. The BEST trial showed the benefit of adding nimotuzumab in locally advanced squamous cell carcinoma of head and neck [12].

Subramanian *et al*. showed promising response rate and survival outcomes by adding nimotuzumab with standard therapy in recurrent and/ or metastatic squamous cell carcinoma of head-and-neck patients without much morbidity [15].

Kumar *et al*. prospectively compared the efficacy and safety of adding nimotuzumab to standard chemoradiotherapy and had shown improved survival rate [16]. Wang *et al*. showed the use of adding nimotuzumab plus chemotherapy with docetaxel, cisplatin, and 5-Fluorouracil in locally advanced recurrent squamous cell carcinoma of head and neck after surgery and radiotherapy and proved the tolerability of the regimen [17].

Yadav *et al*. did a prospective comparative study by adding nimotuzumab to chemotherapy and showed a response rate of 38.2% and median progression-free survival of 5.2 months in the recurrent and metastatic carcinoma of head and neck [18].

Somani *et al*. documented that at 6 months post-treatment with nimotuzumab and chemoradiotherapy, the overall response rate was 80.7%, with 34 patients achieving complete response, and 12 achieving partial response, stable disease in 8 patients, and progressive disease in 3 patients. Nimotuzumab was found to be safe and without serious adverse effects [19].

The side effects observed in this patient were mucositis and neutropenia which were similar to the literature [20]. In addition, this patient also had Grade II pharyngitis which may be attributed to the site of primary disease as well as radiation side effect.

Our patient tolerated the treatment very well when nimotuzumab was added to radiotherapy with minimal side effects.

## Conclusions

Nimotuzumab can be added with radiotherapy to the patients who are ineligible for chemotherapy to improve the clinical outcome with minimal toxicity. Large randomized controlled trials are needed to explore the efficacy and safety of nimotuzumab in chemotherapy ineligible patients.

### Conflicts of interest

The authors declare no conflicts of interest.
